# Presentation and Outcome in S1P-RM and Natalizumab-Associated Progressive Multifocal Leukoencephalopathy: A Multicenter Cohort Study

**DOI:** 10.1212/NXI.0000000000200281

**Published:** 2024-07-11

**Authors:** Julie C. Blant, Nicola N. De Rossi, Ralf Gold, Aude Maurousset, Markus Kraemer, Lucía Romero-Pinel, Tatsuro Misu, Jean-Christophe Ouallet, Maud Pallix Guyot, Simonetta Gerevini, Christos Bakirtzis, Raquel Piñar Morales, Benjamin Vlad, Panajotis Karypidis, Xavier Moisset, Tobias J. Derfuss, Ilijas Jelcic, Guillaume Martin-Blondel, Ilya Ayzenberg, Corey McGraw, David A. Laplaud, Renaud A. Du Pasquier, Raphael Bernard-Valnet, for CORPUS and Italian PML study groups, Alessandra Lugaresi

**Affiliations:** From the Service of Neurology (J.C.B., R.A.D.P., R.B.-V.), Department of Clinical Neurosciences, Lausanne University Hospital (Centre Hospitalier Universitaire Vaudois) and University of Lausanne, Switzerland; Regional Multiple Sclerosis Center (N.N.D.R.), ASST-Spedali Civili di Brescia, Montichiari, Italy; Department of Neurology St. Josef-Hospital (R.G., I.A.), Ruhr University Bochum, Germany; Centre Hospitalier Régional Universitaire de Tours (A.M.), Hôpital Bretonneau, Service de neurologie, Tours, France; Department of Neurology (M.K.), Alfried Krupp von Bohlen und Halbach Hospital, Essen; Department of Neurology (M.K.), Medical Faculty, Heinrich Heine University of Düsseldorf, Germany; Neurology Department (L.R.-P.), Multiple Sclerosis Unit, Hospital Universitari de Bellvitge, IDIBELL, Barcelona, Spain; Department of Neurology (T.M.), Tohoku University Hospital, Japan; Service de Neurologie (J.-C.O.), Pôle des Neurosciences Cliniques, CHU de Bordeaux Pellegrin Tripode; Service de Neurologie et Unité Neurovasculaire (M.P.G.), Centre Hospitalier Régional d'Orléans, France; Unit of Neuroradiology (S.G.), Papa Giovanni XXIII Hospital, Bergamo, Italy; Multiple Sclerosis Center (C.B.), Second Department of Neurology, Aristotle University of Thessaloniki, Greece; Servicio de Neurología (R.P.M.), Hospital Universitario Clínico San Cecilio, Granada, Spain; Department of Neurology (B.V., I.J.), University Hospital Zurich and University of Zurich, ; Neurologic Clinic and Policlinic and Research Center for Clinical Neuroimmunology and Neuroscience (P.K., T.J.D.), Departments of Medicine, Biomedicine, and Clinical Research, University Hospital Basel, University of Basel, Switzerland; Service de Neurologie (X.M.), Université Clermont Auvergne, CHU de Clermont-Ferrand, Inserm, Neuro-Dol; Infectious and Tropical Diseases Unit (G.M.-B.), University Hospital of Toulouse, France; Department of Neurology (C.M.), State University of New York Upstate Medical University, Syracuse; and CHU Nantes (D.A.L.), Service de Neurologie, CRC-SEP, Nantes Université, INSERM, CIC 1413, Center for Research in Transplantation and Translational Immunology, UMR 1064, France.

## Abstract

**Background and Objectives:**

Progressive multifocal leukoencephalopathy (PML) is a severe neurologic disease resulting from JC virus reactivation in immunocompromised patients. Certain multiple sclerosis (MS) disease-modifying therapies (DMTs) are associated with PML risk, such as natalizumab and, more rarely, sphingosine-1-phosphate receptor modulators (S1P-RMs). Although natalizumab-associated PML is well documented, information on S1P-RM–associated PML is limited. The aim of this study is to compare clinical presentations and outcomes between the 2 groups.

**Methods:**

A retrospective multicenter cohort study included patients with PML from 2009 to 2022 treated with S1P-RMs or natalizumab. Data on clinical and radiologic presentation, outcomes, immune reconstitution inflammatory syndrome (IRIS), survival, disability (using the modified Ranking scale—mRS), and MS relapses post-PML were analyzed.

**Results:**

Of 88 patients, 84 were analyzed (20 S1P-RM, 64 natalizumab). S1P-RM–associated PML was diagnosed in older patients (median age 52 vs 44 years, *p* < 0.001) and after longer treatment duration (median 63.9 vs 40 months, *p* < 0.001). Similarly, S1P-RM patients were more prone to show symptoms at diagnosis (100 vs 80.6%, *p* = 0.035), had more disseminated lesions (80% vs 34.9%, *p* = 0.002), and had higher gadolinium enhancement (65% vs 39.1%, *p* = 0.042). Natalizumab patients had a higher IRIS development rate (OR: 8.3 [1.92–33.3]). Overall, the outcome (mRS) at 12 months was similar in the 2 groups (OR: 0.81 [0.32–2.0]). Yet, post-treatment MS activity was higher in S1P-RM cases (OR: 5.7 [1.4–22.2]).

**Discussion:**

S1P-RM–associated PML shows reduced IRIS risk but higher post-treatment MS activity. Clinicians should tailor post-PML treatment based on pre-PML medication.

## Introduction

JC polyomavirus (JCV) is the causative agent of progressive multifocal leukoencephalopathy (PML), a rare opportunistic infection that affects the CNS. In immunocompromised patients, the JC virus may reactivate in the periphery and migrate to the CNS or directly infect the CNS.^1^ Despite PML involving the lytic destruction of oligodendrocytes and extensive demyelination, astrocytes seem to be the primary target of the JC virus.^2^

The escalating use of immunosuppressive treatments of inflammatory diseases has resulted in numerous iatrogenic cases of PML, particularly in patients with multiple sclerosis (MS).^3,4^ Natalizumab (NTZ) has been historically linked to PML since its first report in 2005.^5,6^ Before the introduction of risk management strategies, the incidence was estimated at 2.1 per 1,000 patient-years. However, the implementation of the JCV index in clinical practice significantly decreased the incidence.^7^ In addition, increased awareness and clinical experience with natalizumab-associated PML have contributed to a reduction in the time to diagnosis, a crucial predictor of long-term outcomes.^6^

While natalizumab has been the primary cause of iatrogenic PML in multiple sclerosis, other disease-modifying therapies (DMTs) have been associated, albeit rarely, with JCV reactivation. These include dimethyl fumarate, anti-CD20 agents (rituximab, ocrelizumab), and sphingosine-1-phosphate receptor modulators (S1P-RM; fingolimod, siponimod, ozanimod). To date, 61 patients treated with fingolimod have developed PML, with an estimated incidence rate of 0.0588 per 1,000 patient-years. However, because of its rarity, fingolimod-associated PML remains poorly studied. This presents a challenge with the expanding use of S1P-RM in multiple sclerosis and the aging population under these treatments. Indeed, these new S1P-RM treatments (siponimod, ozanimod) have already been associated with a few cases of PML.^8^

S1P-RM and natalizumab both disrupt T-cell (and to a lesser extent B-cell) trafficking, albeit through different mechanisms. S1P-RM function by antagonizing S1P-R, thereby inhibiting S1P/S1P-R–dependent lymphocyte egress from secondary lymphoid organs. This leads to relative lymphopenia and subsequently reduces T cell infiltration into the CNS, thereby dampening T-cell attack. By contrast, natalizumab is a monoclonal antibody that targets the α4-integrin, a crucial molecule involved in T-cell transmigration to the CNS through α4β1-vascular cell adhesion molecule-1 interaction. By blocking α4-integrin, natalizumab prevents T cells from entering the CNS, thereby reducing inflammation and damage.^9^

The presentation of PML includes a new subacute neurologic deficit associated with supra and infratentorial white matter lesions and/or cortical or cerebellum gray matter lesions.^10^ In contrast to classical PML, natalizumab-associated PML presents at diagnosis with an inflammatory form in up to 40% of cases and is characterized by gadolinium enhancement at diagnosis.^11,12^ Immune reconstitution inflammatory syndrome (IRIS) occurs in almost all cases of natalizumab-associated PML.^13^ Yet, data on fingolimod-associated PML are still limited.^14^ Recurrence of multiple sclerosis activity has been reported after natalizumab-associated and fingolimod-associated PML.^15,16^

Given the limited data available on the course and outcome of S1P-RM–associated PML, we aim to describe it in comparison with cases involving natalizumab.

## Methods

### Design, Settings, and Participants

Patients were identified through a European network of expert centers on PML or from published case reports/series through literature screening on PubMed and Web of Science using the keywords “PML,” “progressive multifocal leukoencephalopathy,” “JC virus,” and “Fingolimod,” “Siponimod,” and “Ozanimod” until October 31, 2023. Corresponding authors were then invited to participate and contribute individual data. In addition, all participating centers were asked to include natalizumab-associated PML encountered in their center (if any).

We conducted a retrospective multicenter cohort study at 39 centers in France, Italy, Germany, Japan, Greece, Spain, the United States, and Switzerland. This study included all consecutive adults (≥18 years) seen at these centers who developed definite, probable, or possible PML according to the 2013 AAN's criteria.^17^ The cases were attributed either to S1P-RM, specifically fingolimod (Gilenya, Novartis) and siponimod (Mayzent, Novartis), or to natalizumab (Tysabri, Biogen) between January 1, 2009, and December 31, 2022. Suspected carryover natalizumab PML while on S1P-RM (≤6 months after natalizumab switch),^18,19^ other JCV-associated clinical presentations (JCV cell granule neuronopathy), and patients with high number of missing values were excluded.

Data were collected using anonymized case report forms (CRF) and centralized using RedCap (Research Electronic Data Capture) software. The CRF gathered data regarding demographics (age, sex), MS history (date of onset, previous DMT, MS activity in the 2 previous years), PML presentation (date and symptoms at onset, MRI features, JCV CSF load, mRS, lymphocyte count), IRIS occurrence (if occurred: date and symptoms at onset, MRI features, JCV CSF load, mRS, lymphocyte count), and PML treatment. Clinical and radiologic evolution for each follow-up, defined by participating centers (including mRS, MRI features, and if performed, JCV CSF load, and lymphocyte count), were also collected. For DMT, IFN-b, glatiramer acetate, teriflunomide, and dimethyl fumarate were classified as immunomodulators and anti-CD20, fingolimod, natalizumab, cyclophosphamide, mitoxantrone, and azathioprine as immunosuppressive therapies (eTable 1). No imputation of missing data was performed. For patients whose disability was not evaluated using the mRS score, a conversion system presented in eTable 2 was used.

### Outcome Measures

The primary outcome was the occurrence of PML-IRIS, defined as clinical deterioration of the PML course following a period of disease stability, associated with signs of immune reconstitution (MRI with gadolinium-enhancing lesions and/or mass effect or inflammatory infiltrate on biopsy). Exploratory variables included short-term and long-term outcomes defined by a value assigned according to the mRS at 12 months and the last follow-up. Survival, disability (defined by an increase ≥2 between the mRS before PML and the last available mRS), radiologic or clinical relapse of MS activity post-PML, and introduction of immunosuppressive therapy were also considered.

### Statistical Analysis

Patient characteristics are expressed as median (interquartile range, IQR 25%–75%) for continuous variables and n (%) for categorical variables, following the Strengthening the Reporting of Observational Studies in Epidemiology guidelines. Comparison across groups was performed using the c^2^ test (categorical variables) and Student t test (continuous variables). *p* values reported were 2-sided, and statistical significance was set at *p* = 0.05. The analysis was conducted using SPSS Statistics 29.0 (IBM SPSS Statistics for Windows, Armonk, NY), R (R Foundation for statistical computing, Vienna, Austria), and Python (Python Software Foundation).

For the analysis of primary and exploratory outcomes, stepwise binomial (survival, IRIS) or ordinal (mRS) logistic regressions were performed by including in the model-independent variables that were considered relevant for their plausible implication on the outcome. We also analyzed IRIS occurrence in a time-dependent manner using a Cox model.

### Artificial Intelligence–Generated Content

ChatGPT 4.0 (OpenAI) has been used for English proofreading and for code generation/correction for R and Python.

### Standard Protocol Approvals, Registrations, and Patient Consents

This study has been approved by the local ethics committee (CER-VD) under the authorization number 2021-01163. A consent waiver was obtained for deceased and lost-at-follow-up patients.

### Data Availability

Anonymized data would be made available upon reasonable request from qualified and noncommercial entities.

## Results

### Baseline Characteristics and Multiple Sclerosis History

**Figure 1:**
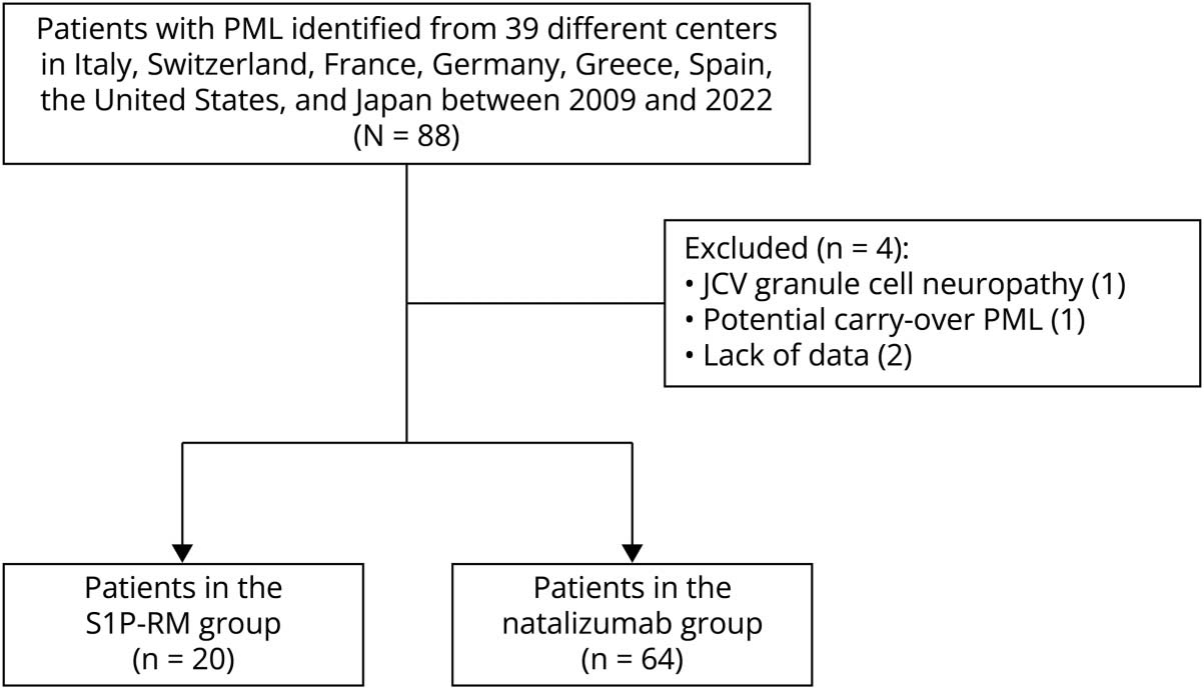
Flowchart

We retrospectively identified 88 patients from 39 international centers across 8 countries (Italy [22 centers, 36 patients], Switzerland [3 centers, 22 patients], France [6 centers, 19 patients], Germany [2 centers, 3 patients], Spain [2 centers, 2 patients], Greece [1 center, 2 patients], the United States [1 center, 2 patients], and Japan [2 centers, 2 patients]) who developed iatrogenic PML between 2009 and 2022. Four participants were excluded: one with carryover natalizumab PML, one with JCV granule cell neuronopathy, and two because of missing data. The final groups consisted of 20 cases of S1P-RM–associated PML and 64 cases of natalizumab-associated PML (Figure 1).

Overall, there were no significant differences between the 2 groups, except that S1P-RM–treated patients were older at PML onset (median (IQR_25-75_): 52 (46.5–60) in S1P-RM vs 44 (38–47) years in NTZ, *p* < 0.001), had a longer MS duration (median (IQR_25-75_): 208.4 (164.2–261) in S1P-RM vs 144 (84–200.4) months in NTZ, *p* = 0.004), and had longer treatment duration (median (IQR_25-75_): 63.9 (46.3–85.9) in S1P-RM vs 40 (26.6–51) months in NTZ, *p* < 0.001) (Table 1). Most patients were female (85% in S1P-RM vs 70.3% in NTZ, *p* = 0.192), with relapsing-remitting course (85% in S1P-RM vs 96.8% in NTZ, *p* = 0.127).

**Table 1:** Baseline Characteristics and PML Course

	S1P-RM (n = 20)	Natalizumab (n = 64)	*p* Value
Baseline characteristics			
Demographic data			
Sex (female), n (%)	17 (85)	45 (70.3)	0.192^a^
Age at PML onset, median (IQR_25-75_)	52 (46.5–60)	44 (38–47)	<0.001^b^
Characteristics of multiple sclerosis (MS)			
Type of MS			0.127^a^
Relapsing-remitting, n (%)	17 (85)	30/31 (96.8)	
Secondary progressive, n (%)	3 (15)	1/31 (3.2)	
Duration of MS before PML onset in months, median (IQR_25-75_)	208.4 (164.2–261)^c^	144 (84–200.4)^d^	0.004^b^
MS activity under DMT in the past 2 years before PML, n (%)	6 (30)	4/21 (19)	0.414^a^
Previous DMT before the one associated with PML			
Previous immunomodulator treatment, n (%)	19 (95.0)	59 (92.2)	0.670^a^
Previous immunosuppressor treatment, n (%)	5 (25.0)	15 (23.4)	0.886^a^
Duration of the treatment associated with PML, median (IQR_25-75_)	63.9 (46.3–85.9)	40 (26.6–51)^e^	<0.001^b^
Baseline EDSS, median (IQR_25-75_)	3 (1.6–5.5)	3.5 (2–5.4)^e^	0.503^b^
Baseline mRS, median (IQR_25-75_)	1.5 (1–3)	2 (1–3)	0.975^b^

Abbreviations: DMT = disease-modifying treatments; IQR = interquartile range; MS = multiple sclerosis; NTZ = natalizumab; S1PR-M = sphingosine-1-phosphate receptor modulators.

a Asymptotic Pearson χ^2^.

b Student *t* test.

c One value is missing.

d Three values are missing.

e Four values are missing.

Prior use of immunomodulating agents (95% in S1P-RM vs 92.2% in NTZ, *p* = 0.67) or immunosuppressive DMT (25% in S1P-RM vs 23.4% in NTZ, *p* = 0.886) was common before introducing the treatment associated with PML. Before PML, the median EDSS was 3 (1.6–5.5) in the S1P-RM group and 3.5 (2–5.4) in the natalizumab group (*p* = 0.503). Similarly, the baseline mRS was comparable in the 2 groups (median (IQR_25-75_): 1.5 (1–3) in S1P-RM vs 2 (1–3) in NTZ, *p* = 0.975) (Table 1 and eTable 1).

### PML Course

PML was more likely to be detected at the asymptomatic stage in NTZ-treated patients (0% in S1P-RM vs 19.4% in NTZ, *p* = 0.035). However, the mean time from the onset of first symptoms to diagnosis did not differ significantly between the 2 groups (median (IQR_25-75_): 15.5 (3.3–88.5) in S1P-RM vs 26 (9.3–69.3) days in NTZ, *p* = 0.636) (Table 2).

**Table 2:** PML Onset and Course

	S1P-RM (n = 20)	Natalizumab (n = 64)	*p* Value
PML characteristics at onset			
Presentation at PML onset, n (%)			0.035^a^
Asymptomatic	0 (0)	6/31 (19.4)	
Acute	6 (30)	3/31 (9.7)	
Progressive	14 (70)	22/31 (71)	
Classification according to PML criteria, n (%)			<0.001^a^
Definite	19 (95)	30 (46.9)	
Probable	0 (0)	21 (32.8)	
Possible	1 (5)	13 (20.3)	
MRI characteristics at onset			
Gd+ lesions (iPML), n (%)	13 (65)	25 (39.1)	0.042^a^
Sub/supratentorial involvement, n (%)			0.098^a^
Subtentorial	3 (15)	8 (12.5)	
Supratentorial	11 (55)	49 (76.6)	
Both	6 (30)	7 (10.9)	
Distribution of PML lesions, n (%)			0.002^a^
Unilobar	1 (5)	21/63 (33.3)	
Multilobar	3 (15)	20/63 (31.7)	
Widespread	16 (80)	22/63 (34.9)	
JC virus load at PML onset			
Copies/mL, median (IQR_25-75_)	852 (106.5–4,923)^c^	294 (29–3,000)^d^	0.205^b^
JCV-negative patients, n (%)	1 (5)	2 (3.1)	0.693^a^
Blood lymphocytes count at PML onset			
Total, median (IQR_25-75_)	365 (295–535)^e^	3,875 (3,190–4,152.5)^f^	<0.001^b^
Time from PML first suspicion to diagnosis in days, median (IQR_25-75_)	15.5 (3.3–88.5)	26 (9.3–69.3)	0.636^b^
PML course and treatment			
Initial attitude			
PML onset during immunosuppressive treatment, n (%)	19 (95)	58 (90.6)	0.537^a^
Delay between PML onset and DMT interruption in days, median (IQR_25-75_)	13 (2–42)^g^	0 (0–14)^h^	0.06^b^
Plasma exchanges, n (%)	5 (25)	51 (79.7)	<0.001^a^
Treatment at the acute phase			
Corticosteroids, n (%)	13 (65)	52 (81.3)	0.129^a^
Context in which corticosteroids were started, n (%)			< 0.001^a^
iPML/IRIS	9 (69.2)	47/47 (100)	
MS relapse	2 (15.4)	0/47 (0)	
Else	2 (15.4)	0/47 (0)	
Maraviroc, n (%)	6 (30)	13/63 (20.6)	0.385^a^

Abbreviations: iPML = inflammatory PML; IQR = interquartile range; IRIS = immune reconstitution inflammatory syndrome; NTZ = natalizumab; S1P-RM = sphingosine-1-phosphate receptor modulators.

a Asymptotic Pearson χ^2^.

b Student *t* test.

c Three values are missing.

d Five values are missing.

e Two values are missing.

f Fifty values are missing.

g One value is missing.

h Seven values are missing.

In the S1P-RM group, on initial MRI presentation, PML lesions were more frequently widespread (80% in S1P-RM vs 34.9% in NTZ, *p* = 0.002) and were more likely to present with inflammatory signs (e.g., with gadolinium-enhancing lesions at the onset—65% in S1P-RM vs 39.1% in NTZ, *p* = 0.042).

Biologically, as anticipated because of the treatment mechanism of action, S1P-RM patients exhibited lymphopenia (median (IQR_25-75_): 365 (295–535) in S1P-RM vs 3,875 (3,190–4,152.5) cells/mm^3^ in NTZ, *p* < 0.001). Yet, the median JC viral load in the CSF was similar in the 2 groups (median (IQR_25-75_): 852 (106.5–4,923) in S1P-RM vs 294 (29–3,000) copies/mL in NTZ, *p* = 0.205) (Table 2).

After the withdrawal of immunosuppressive treatment, the initial management of PML involved plasma exchange in most natalizumab-treated patients (79.7%) and in some S1P-RM–treated patients (25%, *p* < 0.001), although this technique does not remove S1P-RM (Table 2). The use of antiviral treatments (mefloquine, mirtazapine) was frequent but did not differ between the 2 groups.

### Outcome of S1P-RM and Natalizumab-Associated PML

**Figure 2:**
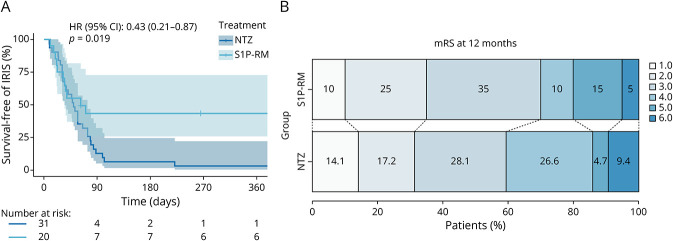
Outcome in S1P-RM and Natalizumab-Associated PML (A) Kaplan-Meier curve of incidence of IRIS over time. HR was obtained through a Cox regression model. (B) Graphical representation of mRS at 12 months (or the last follow-up if ≤12 months). HR = hazard ratio; NTZ = natalizumab; S1P-RM = sphingosine-1-phosphate receptor modulators.

During PML, the proportion of patients developing IRIS was higher in the NTZ group compared with the S1P-RM group (55% in S1P-RM vs 90.6% in NTZ, uncorrected OR: 0.14 [0.04–0.49], *p* = 0.002) (Table 3 and Figure 2A). This difference persisted even after correcting for sex and age at onset (corrected OR: 0.18 [0.04–0.72], *p* = 0.002). However, the mean time elapsed between the interruption of the treatment responsible for PML and IRIS onset was similar in the 2 groups (median (IQR_25-75_): 1.05 (0.62–1.25) in S1P-RM vs 1.57 (0.99–2.41) months in NTZ, *p* = 0.082).

**Table 3:** Outcome Measures Comparing S1P-RM and Natalizumab Patients

	S1P-RM (n = 20)	Natalizumab (n = 64)	Uncorrected OR (95% CI), *p*	Corrected OR (95% CI), *p*
PML-IRIS				
Occurred, n (%)	11 (55%)	58 (90.6%)	0.14 (0.04-0.49) *p* = 0.002	0.18 (0.04–0.72) *p* = 0.002^a^
Outcome				
Short-term outcome mRS at 12 mo, median (IQR_25-75_)	3 (2–4)	3 (2–4)	0.81 (0.32–2.0) *p* = 0.649	0.18 (0.05, 0.67) *p* = 0.01^b^
Long-term outcome mRS at last follow-up, median (IQR_25-75_)	3 (2–4)	3 (2–4)	0.95 (0.38–2.37) *p* = 0.92	0.28 (0.08-0.96) *p* = 0.04^b^
Death ≤12 mo, n (%)	1 (5)	6 (9.4)	0.55 (0.06–4.84) *p* = 0.587	0.54 (0.04–6.8) *p* = 0.451^b^
MS after PML				
MS recurrence after PML, n (%)	16 (80)	27 (42.2)	7.51 (1.99 to 28.33) *p* = 0.003	7.22 (1.73-30.2) *p* = 0.007^c^
Follow-up duration in months, median (IQR_25-75_)	24 (8.7–40)	13.6 (12–34.6)	—	—

Abbreviations: mRS = modified Rankin scale; NTZ = natalizumab; S1P-RM = sphingosine-1-phosphate receptor modulators.

a Corrected for sex and age at PML onset.

b Corrected for sex, age at PML onset, JC virus load, asymptomatic presentation, plurilocular presentation, and mRS before PML.

c Corrected for sex, age at PML onset, previous use of immunosuppressive therapies, and mRS before PML.

These differences in IRIS occurrence resulted in a more frequent use of corticosteroids for IRIS treatment in NTZ-treated patients (69.2% in S1P-RM vs 100% in NTZ, *p* < 0.001). However, the introduction of maraviroc for IRIS prevention and treatment did not differ between the 2 groups (30% in S1P-RM vs 20.6% in NTZ, *p* = 0.385) (Table 2).

Despite the lower rate of IRIS in S1P-RM–treated patients, the outcomes did not differ from those of NTZ-treated patients (Table 3). Neither the 12-month disability (median (IQR_25-75_): 3 (2–4) vs 3 (2–4), uncorrected OR: 0.81 [0.32–2.0], *p* = 0.649) (Figure 2B), disability at last follow-up (median (IQR_25-75_): 3 (2–4) vs 3 (2–4), uncorrected OR: 0.95 [0.38–2.37], *p* = 0.92), nor overall survival (95% in S1P-RM vs 90.6% in NTZ, uncorrected OR: 0.55 [0.06–4.84], *p* = 0.587) showed significant differences (Table 3). Yet, when correcting for independent factors known to affect the outcome, S1P-RM–associated PML had a better outcome at 12 months (corrected OR: 0.18 [0.05, 0.67], *p* = 0.01) and last follow-up mRS (corrected OR: 0.28 [0.08–0.96], *p* = 0.04). In this logistic regression model, the main predictors affecting the outcome were JCV load, symptomatic presentation, and mRS before onset.

### Characteristics of MS Recurrence After PML

After the discontinuation of DMT, MS recurring disease activity was more frequently observed in the S1P-RM group (80% in S1P-RM vs 42.2% in NTZ, uncorrected 7.51 [1.99–28.33], *p* = 0.003, corrected OR: 7.22 [1.73–30.2], *p* = 0.007) (Table 3). Recurring MS activity occurred after a median time of 5 months (median (IQR_25-75_): 4.5 (2.5–10.05) in S1P-RM vs 5 (2.7–14) months in NTZ, *p* = 0.837). Despite being less frequently encountered, recurring activity was associated, in previously NTZ-treated patients, with clinical relapses in most cases (62.5% in S1P-RM vs 96.3% in NTZ, *p* = 0.004) (Table 4).

**Table 4:** MS Recurrence of Disease Activity After PML

	S1P-RM (n = 20)	Natalizumab (n = 64)	*p* Value
MS after PML			
MS recurrence after PML, n (%)	16 (80)	27 (42.2)	**0.003** ^a^
Relapse, n (%)	10/16 (62.5)	26/27 (96.3)	**0.004** ^a^
Radiologic progression, n (%)	15/16 (93.8)	19/23 (82.6)	0.306^a^
Delay between DMT interruption and MS relapse (clinical or radiologic) in months, median (IQR_25-75_)	4.5 (2.5–10.05)	5 (2.7–14)	0.837^b^
Disease-modifying therapies after PML			
Introduction of a DMT after PML, n (%)	12 (60)	34/54 (63)	0.815^a^
Number of DMT introduced after PML, median (IQR_25-75_)	1 (1–2)	1 (1–2)	0.545^b^
Time to introduction of the DMT in months, median (IQR_25-75_)	4.9 (2.8–6.7)	7.3 (5.3–11.8)	0.216^b^
Aggravation of PML post-DMT introduction, n (%)	0/12	0/22	1.0^a^

Abbreviations: DMT = disease modifying therapies; IQR = interquartile range; NTZ = natalizumab; S1P-RM = sphingosine-1-phosphate receptor modulators.

a Asymptotic Pearson χ^2^.

b Student *t* test.

Most patients in both groups resumed treatment (60% in S1P-RM vs 63% in NTZ, *p* = 0.815) after a median time of 6.9 months (median (IQR_25-75_): 4.9 (2.8–6.7) in S1P-RM vs 7.3 (5.3–11.8) months in NTZ, *p* = 0.216) (Table 4). This resumption was mainly under first-line therapies, either platform or oral DMT (eTable 3). A minority of patients had to transition to a more potent DMT during the follow-up (eTable 4). For patients with available data, there was no documented clinical worsening due to PML after DMT reintroduction (Table 4).

## Discussion

The objective of this study was to compare the presentation and outcomes of PML associated with S1P-RM treatments, mainly fingolimod, with those associated with NTZ. Our findings suggest that patients with NTZ-associated PML were more likely to develop IRIS compared with those with S1P-RM–associated PML. Despite these results, there were no significant differences observed in short-term and long-term disability and mortality between both groups. In addition, our study emphasizes the higher risk of resuming MS activity in the aftermath of a PML in S1P-RM than in NTZ-treated patients.

Patients experiencing PML under S1P-RM were older, with longer durations of both MS and treatment. Immune senescence may compromise antiviral immune responses,^20,21^ and aging among MS patients under DMT is associated with an increased risk of infection.^22^ Indeed, aging affects both CD4^+^ and CD8^+^ T lymphocytes, crucial for JCV control, by altering their functionality but also by decreasing the number of naive T cells. These findings align with data published by Novartis on fingolimod-associated PML, revealing that most (59/61) of the patients were treated for more than 2 years. Furthermore, after 6 years of treatment, the risk increased to 17.5 per 100,000 patients per year, compared with an overall risk of 5.88 per 100,000.^23^ Of note, older age was proposed as a risk factor for earlier development of PML and worse prognosis in NTZ-associated PML too.^24,25^

On diagnosis, MRI in S1P-RM–treated patients shows a greater proportion of inflammatory PML lesions (e.g., gadolinium enhancing), potentially indicating a better-preserved immune response. This difference in gadolinium enhancement at the time of PML diagnosis might reflect distinctions in the mechanism of action between NTZ and S1P-RM. Indeed, despite lymphopenia, S1P-RM–treated patients can mount T-cell responses, suggesting that under infectious conditions T cells may egress from lymph nodes using alternative cues than S1P-R.^26^

These differences in the kinetic of the CNS immune reconstitution may also explain the different rates of IRIS in the 2 groups. Indeed, after fingolimod discontinuation, it has been demonstrated that early immune reconstitution remains partial.^21^ Normalization of T-cell subsets may take several months and occur after the total lymphocyte count and CD4:CD8 ratio have returned to baseline.^21^ Such delayed recovery of the lymphocyte function may explain why IRIS is less frequent or blunted. On the contrary, plasma exchanges, used in most NTZ cases in our work, forcing rapid immune restoration may cause IRIS and worsen the prognosis by prolonging IRIS duration.^27^

In PML associated with immunosuppressive treatment, IRIS was suggested in MS to be associated with a poorer outcome.^6,13,27^ Consequently, we initially hypothesized that short-term and long-term disability would be better in patients receiving S1P-RM. However, our crude results contradict this hypothesis. Yet, by correcting for factors suggested to be associated with PML severity (sex, age at PML onset, JC virus load, asymptomatic presentation, plurilocular presentation, mRS before PML), we have shown that the outcome was better in S1P-RM–associated PML. It highlights that the prognosis of S1P-RM PML was hindered by a delayed detection with all cases already symptomatic and with widespread lesions. Indeed, it can probably be explained by the fact that NTZ-treated MS patients were more closely radiologically monitored than the S1P-RM–treated ones.^28^ Interestingly, the recurrence of MS was found to be more common among patients using S1P-RM compared with those with NTZ-associated PML cases. It remains to be determined whether this recurrence could have contributed to explaining the relatively poor overall prognosis.

Both S1P-RM and NTZ are treatments associated with recurring disease activity after withdrawal.^29,30^ However, the rate of S1P-RM resuming MS activity was surprisingly high, affecting 80% of our patients with PML, compared with approximately 30% after withdrawal of fingolimod in the general population with MS.^29,31^ Nevertheless, the percentage of patients experiencing recurring activity was comparable with those who discontinued S1P-RM without transitioning to another DMT.^29^ In our cohort, the median delay of 4 months before recurring activity was in line with previous studies analyzing patients without PML. Of note, contrary to NTZ-treated patients who experience mostly clinical relapses, about one-third of these recurrences were only radiologic in SP1-RM–associated PML.

Resumption of MS DMT took place in 60% of all patients after PML stabilization whether the activity of MS was recurring. Initial preferences leaned toward either platform-based options (IFN-β, Glatiramer acetate) or oral medications (dimethyl fumarate, teriflunomide). As previously demonstrated, the reintroduction of DMT, typically occurring after a median time of 6.9 months, was not associated with a recurrence of PML activity,^32^ suggesting that the JCV-specific immune response had time to clear the virus from the brain.^33,34^ In addition, tissue-resident memory T cells may establish enduring immune control against JCV, which is minimally affected by DMT.^35^

Several study limitations should be acknowledged, such as its retrospective design, and the variations in prevention and follow-up protocols across different countries. Limited information on factors such as annualized relapse rate and lymphopenia kinetics hinders a precise evaluation of the risk factors associated with PML development in S1P-RM–associated cases.

It is crucial to note that the distribution of cases between the 2 groups is not uniform over time. Most NTZ cases are dated back to the early 2010s before the implementation of risk-mitigating strategies with JCV antibody index detection before NTZ introduction.^7^ By contrast, S1P-RM–associated PML cases, with the first reported instance in 2015, are more recent.^14^ This temporal difference may have influenced the care provided to these patients, potentially affecting the comparison of treatment strategies for PML or MS and, consequently, the overall outcomes.

Nevertheless, this study defines the characteristics of S1P-RM–associated PML and compares those with the better-known NTZ-associated PML. Altogether, our results underscore the distinctions in clinical presentation and progression between PML associated with NTZ and S1P-RM. These variations emphasize the necessity of tailoring PML management based on the specific therapy used.

This consideration is equally applicable to the management of MS following treatment withdrawal. Clinicians should consider early reintroduction of DMT to avoid MS recurrence, which could contribute to worsening the prognosis. Our data suggest that once an anti-JCV response is established, it would be safe to treat MS appropriately. However, current data are too scarce to detail the type of DMT and the timing of reintroduction that should be advised.

Furthermore, our results indicate that PML associated with S1P-RM was detected at a later stage, with no asymptomatic cases and a higher rate of widespread lesions. This emphasizes the need for increased awareness among neurologists and suggests that closer clinical-radiological surveillance may be warranted for older patients under S1P-RM treatments. The role of other biomarkers, such as the JCV index,^36^ remains elusive, although there have been suggestions that it could be of interest, particularly in older patients.^37^ However, it is worth noting that most patients in this study did not undergo JCV index testing before PML.

Further studies are imperative to precisely delineate the risk of PML associated with S1P-RM, particularly in older patients, and to explore potential biomarkers that could predict PML development under these treatments. However, such analyses are currently limited by the number of reported cases. Nevertheless, collaborative efforts are needed to find ways to implement effective risk-mitigating strategies, similar to those used for natalizumab-treated patients.^1,7^

## Appendix 1 Authors

**Table:**

Name	Location	Contribution
Julie C. Blant, MD	Service of Neurology, Department of Clinical Neurosciences, Lausanne University Hospital (Centre Hospitalier Universitaire Vaudois) and University of Lausanne, Switzerland	Drafting/revision of the manuscript for content, including medical writing for content; analysis or interpretation of data
Nicola N. De Rossi, MD	Regional Multiple Sclerosis Center, ASST-Spedali Civili di Brescia, Montichiari, Italy	Drafting/revision of the manuscript for content, including medical writing for content
Ralf Gold, MD	Department of Neurology St. Josef-Hospital, Ruhr University Bochum, Germany	Drafting/revision of the manuscript for content, including medical writing for content
Aude Maurousset, MD	Centre hospitalier régional universitaire de Tours, Hôpital Bretonneau, Service de neurologie, Tours, France	Drafting/revision of the manuscript for content, including medical writing for content
Markus Kraemer, MD	Department of Neurology, Alfried Krupp von Bohlen und Halbach Hospital, Essen; Department of Neurology, Medical Faculty, Heinrich Heine University of Düsseldorf, Germany	Drafting/revision of the manuscript for content, including medical writing for content
Lucía Romero-Pinel, MD, PhD	Neurology Department, Multiple Sclerosis Unit, Hospital Universitari de Bellvitge, IDIBELL, Barcelona, Spain	Drafting/revision of the manuscript for content, including medical writing for content
Tatsuro Misu, MD, PhD	Department of Neurology, Tohoku University Hospital, Japan	Drafting/revision of the manuscript for content, including medical writing for content
Jean-Christophe Ouallet, MD, PhD	Service de Neurologie, Pôle des Neurosciences Cliniques, CHU de Bordeaux Pellegrin Tripode, France	Drafting/revision of the manuscript for content, including medical writing for content
Maud Pallix Guyot, MD	Service de Neurologie et Unité Neurovasculaire, Centre Hospitalier Régional d'Orléans, Orléans, France	Drafting/revision of the manuscript for content, including medical writing for content
Simonetta Gerevini, MD	Unit of Neuroradiology, Papa Giovanni XXIII Hospital, Bergamo, Italy	Drafting/revision of the manuscript for content, including medical writing for content
Christos Bakirtzis, MD, PhD	Multiple Sclerosis Center, Second Department of Neurology, Aristotle University of Thessaloniki, Greece	Drafting/revision of the manuscript for content, including medical writing for content
Raquel Piñar Morales, MD	Servicio de Neurología, Hospital Universitario Clínico San Cecilio, Granada, Spain	Drafting/revision of the manuscript for content, including medical writing for content
Benjamin Vlad, MD	Department of Neurology, University Hospital Zurich and University of Zurich, Switzerland	Drafting/revision of the manuscript for content, including medical writing for content
Panajotis Karypidis, MD	Neurologic Clinic and Policlinic and Research Center for Clinical Neuroimmunology and Neuroscience, Departments of Medicine, Biomedicine, and Clinical Research, University Hospital Basel, University of Basel, Switzerland	Drafting/revision of the manuscript for content, including medical writing for content
Xavier Moisset, MD, PhD	Service de Neurologie, Université Clermont Auvergne, CHU de Clermont-Ferrand, Inserm, Neuro-Dol, France	Drafting/revision of the manuscript for content, including medical writing for content
Tobias J. Derfuss, MD	Neurologic Clinic and Policlinic and Research Center for Clinical Neuroimmunology and Neuroscience, Departments of Medicine, Biomedicine, and Clinical Research, University Hospital Basel, University of Basel, Switzerland	Drafting/revision of the manuscript for content, including medical writing for content
Ilijas Jelcic, MD	Department of Neurology, University Hospital Zurich and University of Zurich, Switzerland	Drafting/revision of the manuscript for content, including medical writing for content
Guillaume Martin-Blondel, MD, PhD	Infectious and Tropical Diseases Unit, University Hospital of Toulouse, France	Drafting/revision of the manuscript for content, including medical writing for content
Ilya Ayzenberg, MD	Department of Neurology St. Josef-Hospital, Ruhr University Bochum, Germany	Drafting/revision of the manuscript for content, including medical writing for content
Corey McGraw, MD	Department of Neurology, State University of New York Upstate Medical University, Syracuse	Drafting/revision of the manuscript for content, including medical writing for content
David A. Laplaud, MD, PhD	CHU Nantes, Service de Neurologie, CRC-SEP, Nantes Université, INSERM, CIC 1413, Center for Research in Transplantation and Translational Immunology, UMR 1064, France	Drafting/revision of the manuscript for content, including medical writing for content
Renaud A. Du Pasquier, MD	Service of Neurology, Department of Clinical Neurosciences, Lausanne University Hospital (Centre Hospitalier Universitaire Vaudois) and University of Lausanne, Switzerland	Drafting/revision of the manuscript for content, including medical writing for content; study concept or design
Raphael Bernard-Valnet, MD, PhD	Service of Neurology, Department of Clinical Neurosciences, Lausanne University Hospital (Centre Hospitalier Universitaire Vaudois) and University of Lausanne, Switzerland	Drafting/revision of the manuscript for content, including medical writing for content; major role in the acquisition of data; study concept or design; analysis or interpretation of data

## Appendix 2 Coinvestigators

**Table:**

Coinvestigators are listed at Neurology.org/NN.

## Glossary

CRF: case report forms
DMT: disease-modifying therapy
IRIS: immune reconstitution inflammatory syndrome
JCV: JC polyomavirus
mRS: modified Ranking Scale
MS: multiple sclerosis
PML: progressive multifocal leukoencephalopathy
S1P-RMs: sphingosine-1-phosphate receptor modulators
